# Conscientious objection to abortion, the law and its implementation in Victoria, Australia: perspectives of abortion service providers

**DOI:** 10.1186/s12910-019-0346-1

**Published:** 2019-01-31

**Authors:** Louise Anne Keogh, Lynn Gillam, Marie Bismark, Kathleen McNamee, Amy Webster, Christine Bayly, Danielle Newton

**Affiliations:** 1 Centre for Health Equity, Melbourne School of Population and Global Health, The University of Melbourne, Melbourne, Victoria 3010 Australia; 2 Children’s Bioethics Centre, Royal Children’s Hospital, Melbourne, Australia; 3 Centre for Health Policy, Melbourne School of Population and Global Health, The University of Melbourne, Melbourne, Australia; 4 Family Planning Victoria, Melbourne, Australia; 5 Department of Obstetrics and Gynaecology, Monash University, Clayton, Australia; 6 Women’s Health Victoria, Melbourne, Australia; 7 The Royal Women’s Hospital, Melbourne, Australia

**Keywords:** Abortion, Conscientious objection, Qualitative methods, Regulation, Health services, Law reform

## Abstract

**Background:**

In Victoria, Australia, the law regulating abortion was reformed in 2008, and a clause (‘Section 8’) was introduced requiring doctors with a conscientious objection to abortion to refer women to another provider. This study reports the views of abortion experts on the operation of Section 8 of the Abortion Law Reform Act in Victoria.

**Methods:**

Nineteen semi-structured qualitative interviews were conducted with purposively selected Victorian abortion experts in 2015. Interviews explored the impact of abortion law reform on service provision, including the understanding and implementation of Section 8. Interviews were transcribed verbatim and analysed thematically.

**Results:**

The majority of participants described Section 8 as a mechanism to protect women’s right to abortion, rather than a mechanism to protect doctors’ rights. All agreed that most doctors would not let moral or religious beliefs impact on their patients, and yet all could detail negative experiences related to Section 8. The negative experiences arose because doctors had: directly contravened the law by not referring; attempted to make women feel guilty; attempted to delay women’s access; or claimed an objection for reasons other than conscience. Use or misuse of conscientious objection by Government telephone staff, pharmacists, institutions, and political groups was also reported.

**Conclusion:**

Some doctors are not complying with Section 8, with adverse effects on access to care for some women. Further research is needed to inform strategies for improving compliance with the law in order to facilitate timely access to abortion services.

## Background

Abortion is regarded by major international health organisations as a simple and safe medical procedure, forming an essential part of reproductive health services [1]. However, many countries allow doctors to refuse to provide abortion services if they have a moral or religious objection to doing so [2]. This puts medical provision of abortion in an unusual situation, and one that unfairly impacts women. In most fields of medicine a doctor may not refuse to help a patient access a service which is legally permitted, efficient, and beneficial to the care of a patient simply because it conflicts with their values [3]. For example, a surgeon who has a religious objection to blood transfusion cannot use that as grounds for denying a patient access to a major surgical procedure that may require transfusion.

Although conscientious objection (CO) is often linked to religious freedom [4, 5], one key ethical justification for permitting CO is the value of personal moral integrity [6, 7]. Brock argues that moral integrity needs to be protected because deeply held moral commitments are a central part of personal identity [6]. However, as Brock also points out, the value of integrity is one value among a number, and can come into conflict with other important values. These other values, notably obligations to provide help to patients when acting in the role of a health professional, can be overriding. The most commonly accepted resolution of this ethical conflict, Brock [6] calls the “conventional compromise” and Minerva [4] describes as the “moderate position”. It aims to allow scope for CO but only in ways that do not significantly undermine women’s health or right to access services.

The usual conditions placed on conscientious refusal to perform or provide information about abortion are clearly articulated by Brock [6]. Most importantly, CO is only legitimate in circumstances where it does not impose an unreasonable burden on the patient (in terms of delay or distress or health consequences). When this condition is met, a professional may declare their CO and decline to provide a service, but must inform the patient that abortion is an available service, and refer the patient to another professional who is able to provide the service. This is the position of professional bodies, including the World Medical Association [8], the International Federation of Gynaecology and Obstetrics [9], and the Royal Australian and New Zealand College of Obstetricians and Gynaecologists [10].

In terms of what is legally permitted, there is variation globally in the conditions or limits which are placed on CO to abortion. At one end of the spectrum are countries including Sweden, Finland, Bulgaria, Czech Republic and Iceland [2, 11] which do not permit CO. This position is established through a combination of law, policy and practice, for example, in Sweden [11], the abortion act is a rights-based law, there is a policy ban on CO, abortion care is an essential component of medical training, provision of abortion in public hospitals is expected, and ‘those who object to performing abortions cannot become obstetricians/gynaecologists or midwives’ (p2). In addition, legal challenges to the CO ban have so far failed [2]. Some countries take the ‘moderate position’ or ‘conventional compromise’ described earlier [7], allowing CO but imposing a range of conditions designed to reduce the impact of the objection on women’s access to services. Italy, for example, requires doctors to register their objection in writing. Many other countries do not require registration [12], but do impose some version of an obligation to refer the patient to another provider. Other countries, like Poland, are closer to the “conscience absolutism” end of the spectrum, meaning doctors neither have an obligation to provide care that conflicts with their conscience nor any obligation to facilitate access to care by another provider [7]. The global variation in legal CO provisions is partly due to differing weights ascribed to the two competing values described above, which can be framed in terms of the competing rights of health professionals and women [4].

Victoria, a state of Australia with a population of around 6 million, has adopted the conventional compromise position, as the refusing doctor is required to refer the woman to a doctor who does not have a CO to abortion [13]. However, doctors are not required to register or justify their objection. Globally, concern has been expressed about potential misuse or abuse of CO provisions, particularly in countries where there is opposition to recent liberalisation of abortion laws (eg Colombia) or pressure to re-criminalise abortion (e.g. Poland) [14]. Meyers and Woods [15] argue that one of the problems with CO in California was the process of declaring CO was ‘so simplistic as to trivialise moral decision-making’ (p117). Where there are no regulations to ensure that conscientious objectors genuinely have deeply and consistently held moral or religious positions, permitting CO may result in unjustified restriction of access to abortion services [14, 16] However, there is little research on how CO operates in practice in different legal settings, and what effects it has on women’s access and experience.

Data on the rates of CO to abortion are often poor quality, and data on the practice of those holding a CO is limited. Where reported, rates of CO vary from 15% of health care professionals in Australia to up to 70% in Italy and Poland [2, 12, 17). A survey of over one thousand US physicians revealed that 52% of the sample objected to abortion. In contrast, a survey of Australian obstetrics and gynaecology fellows and trainees found that only 15% of the 740 participants held views that made them totally opposed to abortion [17]. Data about how health professionals act when they do have a CO, or when they work in a jurisdiction which makes CO easy to espouse, is also limited. Curlin and colleagues [18] found that of US physicians who objected to abortion, only 60% believed they should be obliged to refer the patient. French and colleagues [19] surveyed clinicians’ referral practices for a range of conditions and found that only 52% of 496 participants indicated they had a professional obligation to refer in the case of abortion. Analysis of qualitative comments revealed only 18% would facilitate a referral, while 39% would provide ‘just-the-name’ of a clinic or doctor, 29% would offer nothing and 15% would provide misleading information [20]. Similar figures are reported by Holt and colleagues [21] who also report that 14% of US primary care physicians routinely attempt to dissuade women from abortion. These US studies suggests that a significant proportion of doctors who claim a CO may not be meeting professional obligations to refer. Doctors’ practice in Victoria, Australia, where the obligation to refer is enshrined in law, is poorly understood.

### Legal setting in Victoria, Australia

In 2008, the Victorian Parliament passed the Abortion Law Reform Act (Vic.) [13] with the stated intention of bringing the law relating to termination of pregnancy into line with current practice and community attitudes. Given abortion was available in public and private facilities in Victoria and expected by women, the purposes of this Act were threefold: to remove abortion from the Crimes Act 1958; to clearly specify the grounds on which abortion may take place; and to outline the obligations of registered health practitioners with a CO to abortion. Under this Act, abortion became legally permissible for a woman giving free and informed consent at any stage of gestation and for any reason, with the proviso that after 24 weeks, two doctors must agree that “it is appropriate in all the circumstances” [13]. Despite this law reform, access remains restricted in practice [22], particularly in rural areas [23].

Section 8 of the Act states that any health practitioner who is asked to advise a woman about abortion, or perform, direct, authorize or supervise an abortion, and who has a CO to abortion must: 1) inform the woman that they have a CO; and 2) refer the woman to another health practitioner, in the same profession, who the practitioner knows does not have a CO to abortion. Non-compliance with the guidelines set out in Section 8 may result in charges of professional misconduct by the practitioner’s registering authority.

Despite this legal obligation being aligned with international and national medical guidelines and codes, Section 8 has proved to be a controversial addition to the Act [24–27]. Opponents of Section 8 argue it compromises practitioners’ religious or moral stance on abortion by compelling those with a CO to be complicit in allowing access to abortion through the act of referral [28–30]. In Victoria, groups like “Doctors in conscience” have advocated for Section 8 to be repealed [31, 32]. Against this, those who support an ‘obligation to refer’ by practitioners who are unwilling to provide the service themselves believe it is necessary to ensure that women are able to access an abortion with minimal disruption to their care [28, 33, 34].

Given the degree of controversy associated with practitioner obligations set out in Section 8, and concerns globally about the impact of such provisions on access to abortion services, it is surprising that little empirical research has been undertaken on the practice of CO. Through the perspectives of experts on abortion services in Victoria, this study aimed to explore health professionals’ understandings of the inclusion of Section 8 in the Abortion Law Reform Act, as well as their perceptions of how Section 8 has been implemented in the Victorian health system and its impact on care.

## Methods

Given the paucity of information on the use of CO since the law reform in Victoria in 2008, we adopted a constructivist paradigm, and used qualitative methodology, as both are suited to initial exploratory research. Exploration of CO was included in a broader study designed to investigate the effects of abortion law reform in Victoria [22]. Experts in abortion provision were considered best positioned to comment on the impact of CO on service delivery. We considered an individual an ‘expert’ if they were involved directly in the provision of either medical or surgical abortion, provided counselling in relation to accessing abortion, or were involved in policy or advocacy related to abortion access. Experts were identified through researcher networks and snowball sampling was used to expand the sample. Purposive sampling was used to select participants with the most knowledge, expertise, and clinical experience in the area of abortion service provision. We also purposively sampled to ensure we included experts affiliated with a variety of health organisations in a range of geographical locations. Nineteen experts were invited to take part in the study by email, and all agreed to participate (See Table 1). At the time of interview, 15 experts were employed by an organisation providing medical abortion, and 10 experts were employed by an organisation providing both surgical and medical abortion.

**Table 1:** Key informant characteristics (*n* = 19)

Characteristic	Number
Gender	
Female	15
Organisation*	
Sexual and reproductive health service	7
Hospital	
*Public Hospital*	5
*Private Hospital*	1
General Practice	3
Community health service	2
Reproductive health service	2
Sexual health service	1
Young person’s health service	1
Professional Role	
General Practitioner	5
Obstetrician & Gynaecologist	4
Medical Practitioner	3
Service Manager	3
Primary Health Care Nurse	2
Psychologist	1
Sexual Health Physician	1
Geographical location	
Metropolitan	11
Regional	8

*participants were able to indicate they worked for more than one organisation

We developed a semi-structured interview schedule to explore with experts a range of issues related to abortion provision [22, 35, 36]. For part of the interview, we explored perceptions of Section 8 of the Abortion Law Reform Act and how it was being implemented by health care providers in the Victoria at the time of the interview (2015). There was scope during the interview for participants to raise additional unanticipated issues. Interviews were conducted by experienced qualitative researcher DN (PhD), a research fellow employed on the study, either face-to-face or over the telephone and the whole interview lasted between 30 min and 2 h (only part of which was spent on Section 8); de-identified audio-recordings were transcribed verbatim.

Based on the interview schedule and the identification of any new themes emerging from the data, a coding framework was developed. Data related to Section 8 was initially coded by LK, and a sample of transcripts was double coded by DN to ensure reliability of interpretation of codes. Thematic analysis of the coded data was then undertaken by LK using the method of constant comparison to develop categories in each theme in order to fully explain the variation present in the data [37]. DN checked the categorisation of the coded data and any discrepancies were discussed and a mutually agreeable interpretation was reached. Data analysis was managed in word processing software.

## Results

First, we present experts’ understanding of the purpose and value of Section 8, and their perspectives on the intent of enshrining it in law. Secondly we detail participants’ descriptions of how Section 8 is implemented in health and medical practice, including the categories of misuse reported.

### Purpose of section 8

When asked to describe the Section 8 provisions, all participants were aware of Section 8 of the Abortion Law Reform Act and were able to describe the general premise of Section 8. For example,

*‘My understanding of the clause is that if you have a conscientious objection to providing advice or services around termination of pregnancy, as a medical practitioner you are obliged under the law to refer the patient to a colleague who doesn’t have that conscientious objection.’* {8}

### Section 8 perceived as a mechanism to ensure women’s rights

When describing the clause, the majority of participants focused on the doctor’s obligation to refer, and saw this as an essential requirement to ensure women were not denied access to a service they were legally entitled to. All participants were supportive of Section 8 as it was seen to be concerned with ensuring women receive optimal care irrespective of practitioners’ personal, moral or religious views on abortion.

*‘Medicine should be about providing care for the patient not providing care for the doctor.’ {8}*

*‘No matter what your thoughts are, it is not your responsibility to pass judgment on someone else, and you know if someone does not want to do a termination, that’s their choice, but their medical responsibility to transfer care of the patient to someone who is prepared to give them appropriate treatment.’ {4}*

*‘Yeah I'm happy for people to be able to have a conscientious objection as long as they maintain their duty, which is you know getting people through pretty quickly, yeah.’ {13}*

### Section 8 perceived as a mechanism to protect doctor’s rights

Far fewer participants focused on Section 8 as a mechanism to protect doctor’s rights. Only a few emphasised the importance of practitioners not being forced into providing a service that goes against their conscience. Even those who were sympathetic to protecting doctors in this way still emphasized the importance of referral. For example,

*‘So I think people should be allowed to – if something is deeply against their conscience they should be allowed to not be involved, but they have to acknowledge it, be prepared to rationalise it and be able to articulate it and refer on. I really do think that the referral on is really important’ {12}*

### Does it need to be enshrined in law?

Participants were divided as to how important it was that the responsibilities of conscientious objectors be enshrined in law. Some felt the underlying premise was similar to the principles outlined in the Australian Medical Association’s Code of Ethics [38] and the Medical Board of Australia’s ‘Good medical practice: a code of conduct for doctors in Australia’ [39], and therefore wondered why Section 8 had been positioned in law rather than specifically embedded within professional practice guidelines.

*‘I've spoken to some people about why and I'm not sure I've completely got an answer like why it needs to be in law rather than in a professional practice sort of standing. But I do think it's extremely important’ {3}*

Other participants valued having the CO process clearly defined in law, making practitioner obligations clear. In particular, it was seen to provide clarification for those practitioners employed within health services that have chosen to ‘opt-out’ of abortion. Section 8 ensures that these practitioners are required to honour their individual *legal* responsibility to ensure women receive care irrespective of the views of the service they work for.

*‘It makes an enormous difference to have it in law - I think this is a big deal, to have in law that doctors are legally bound to refer you on to a service that will provide the service. It’s not necessarily going to happen just like that, but to have that clear is really good’ {10}*

### Perceptions of how section 8 was implemented in medical practice

There was a strong feeling that most doctors would not let their moral or religious beliefs impact on the care they provide to their patients, and all acknowledged that for the majority of doctors working in Victoria, adhering to the law was common sense and worked well,

*‘I think most doctors do practice their medicine for their patients rather than for their own conscientious or religious beliefs’ {8}*

However, all participants could describe negative consequences related to the practice of CO in Victoria, and these were perceived to have occurred even after the clarity provided by law reform in 2008. The negative consequences arose either from section 8 not being followed, or being followed inappropriately. All nineteen participants were able to relate specific stories about doctors subverting, misusing or directly contravening the law.

### Doctors directly contravening the law by not referring

It was common for participants to report instances of doctors directly contravening Section 8, by not referring women seeking abortion to someone who could advise them. Not only was it commonly reported by participants, but some participants working in rural areas described refusal to refer as a ‘common practice.’

*‘Women tell us that GPs not only won’t assist them but they won’t refer them on either. We think there are problems in enacting that law….’ {1}*

*‘Yes, I don’t know if it’s known very much. I don’t know if it’s gone out to doctors really. Certainly women come to me having had doctors be very rude to them and not necessarily refer them on anywhere ... Yes there are quite a few conscientious objectors in our town and they’re not nice to the girls particularly at all. Things such as, “No, I don’t do that”, and then just standing up and opening the door for the patients to leave. That’s very, very common here certainly, and I don’t know how much that’s been – I don’t think the doctors know that’s a law among the conscientious objectors’ {6}*

*‘See, we sometimes get women saying, “I got told that I wouldn’t get an abortion,” because the women don’t know all the ins and outs of the law and some of the rural women say that the only information they got was that, “you’re too far on” or, “you won’t get a service” or, “we don’t deal with that here, go somewhere else”’ {12}*

In rare cases, participants were aware of incorrect advice from a doctor resulting in a woman not being able to arrange a termination, and being forced to continue a pregnancy that she was seeking to terminate.

*INT Have you heard any anecdotal evidence from women about their experience perhaps outside of your service with conscientious objectors?*

*13 I have. So two in particular for late termination. Yeah, and … both these women were, it was the same practitioner, completely misinformed unfortunately …*

*INT Were there any negative repercussions for the women involved in those situations?*

*13 Yeah well one was certainly able to still seek termination, and one wasn’t, one continued…*

### Doctors attempting to delay women’s access

Not all participants reported doctors directly contravening the law by refusing to refer women. Many instead reported they knew of instances of doctors trying to deter women from having an abortion, or of doctors purposely delaying women to make accessing abortion more difficult.

*‘Well we still get some patients coming in and saying “oh gee, I went to my doctor and he was not too helpful, and sent me on the run-around waiting for this ultrasound, and then come back and see me a week later and on and on,” we still get that. That hasn’t changed since the Law Reform, but I think we are getting more patients referred to other doctors within a particular practice, if that doctor has a conscientious objection to it.’ {9}*

*‘I’d say it would be very common for me … that I would hear stories of - I don’t know whether it would be one in 20, it’s very hard to put a figure on it, of women who struggled, who went to their GP first to find out where they could go, and where the GP clearly didn’t agree, and may not have necessarily stopped them from getting the information about where to go, because it is pretty easy to find out if you just turn on your computer. But definitely was trying to get them to change their mind… to deter them or delay them.’ {10}*

### Doctors attempting to make women feel guilty

Other participants described situations in which women were not prevented from accessing abortion services or deliberately delayed, but instead were made to feel guilty about requesting an abortion. Participants pointed out that women already feel guilt, and some participants thought this behaviour, likely to increase the distress of someone young and vulnerable, was immoral.

*‘The way it's done could be extremely damaging to someone who might ultimately easily access the service anyway.’ {3}*

*‘They’re made to feel guilty about it … They feel guilty anyway … There’s no need to you know - there’s no need to push it into their faces, and I think that’s what sometimes happens.’ {4}*

*‘The other thing that I guess I'm concerned about too with doctors telling a woman that they have a conscientious objection … it's so judgmental to that woman. If a woman's pregnant whether she wants to be or doesn't want to be, often she's in a more vulnerable state and to have someone in authority like that, who she's dependent on for care at that moment to say, “what you're asking for I think is wrong and is immoral,” I think it's a slap in the face to that woman.’ {5}*

### Doctors objecting for reasons other than conscience

There were concerns among several participants that some doctors see the recognition of CO in Section 8 as legitimizing a choice to opt out of abortion service provision. They expressed frustration that doctors who felt it would be easier not to be involved in abortion service provision, or for whom there might be reputational penalties could claim a CO, even if they did not hold a religious or moral position incompatible with providing abortion.

*‘You just say, “I’m not doing them.” You don’t have to discuss it or justify it…So what’s happening here is not conscientious objection. It’s just ‘opt out’.’ {12}*

*‘I think there needs to be limits, you know conscientious objection can’t be an unlimited thing that anybody who just doesn’t want touch something in the slightest way… you think of all the areas that you’re not allowed to conscientiously object but you can here, and everyone can, at the drop of a hat.’* {10}

### Use of conscientious objection by individuals and groups other than doctors

In addition, but less commonly, participants described unintended consequences from CO that occurred in contexts other than doctor’s direct provision of care to their patients. There were instances of claims being made by individuals other than doctors, or by institutions rather than individuals. In addition, they described the misuse of the CO clause by political groups.

### Telephone staff

Some described telephone staff in government services refusing to be involved in access to medical abortion;

*Not an uncommon experience for me … to ring up Canberra to get authority under PBS* [Pharmaceutical Benefits Scheme] *to use the medication between five and seven weeks … not uncommon for the person on the other end of the phone to say “I’m sorry, I will not,” you know, I mean the person will just say “I will not have my hand in this process of you giving that medication to that woman.” Now it wouldn’t happen a lot, but I’d probably say it has happened about six times to me. {10}*

### Pharmacists

Some described pharmacists refusing to stock medication related to abortion;

*‘We’ve got one pharmacist in town who won’t even give out the pill, so that’s quite problematic.’* {11}

### Institutions

Another related concern expressed by some participants was an objection to institutions such as private or Catholic hospitals using the clause to endorse their right to ‘opt out’ of providing abortion services. This was the case even for maternity hospitals providing prenatal genetic testing services. Genetic testing service models are predicated on the notion that an option for women found to have certain fetal abnormalities is termination of the pregnancy. Several participants expressed concern about this practice.

“*…I have a really strong objection to institutional opting out because there’s nothing in the law about that and that’s wrong” {12}*

### Political groups

A few participants noted that the CO clause in legislation is used politically by anti-abortion groups to undermine law reform. These groups were reported to have used the media to fuel the perception that Section 8 forces practitioners who conscientiously object to refer women to a service where they can obtain an abortion. In reality, a practitioner’s obligation is simply to refer women to another practitioner “in the same regulated health profession who the practitioner knows does not have a conscientious objection to abortion.” So, in practice, a general practitioner only needs to refer to another general practitioner who does not have a CO to abortion, not to an abortion service. While this may cause moral conflict for some, participants expressed concern that the message promoted by anti-abortionists could provoke unnecessary concern for GPs who may otherwise be willing to refer women to another GP.

*‘It’s seen as a focal point from the anti-abortionists, that this is something they can jump up and down about and say “oh no, well we can’t refer somebody for an abortion.” But that’s not what it’s requiring you to do, it’s requiring you to send them to somebody who will discuss all the alternatives’ {19}*

## Discussion

The increasing specialisation of modern medicine means many doctors only provide some of the services that fall within their scope of practice. However, where a doctor is not willing or able to provide a particular service, they have an ethical obligation to refer the patient to someone who may be able to assist. In effect, section 8 of the Abortion Law Reform Act formalises that professional expectation in relation to abortion by imposing a legal obligation on doctors with a CO to abortion to refer women to someone without such an objection. This compromise is designed to allow for the moral integrity of the doctor, but only so far as this can be maintained while not causing harm to patients. On the basis of our findings, we argue that the compromise has not always been maintained by doctors in Victoria, and that the practice of CO to abortion can cause damage to patients, ranging from delay or distress through to serious health consequences for women. We argue there are two key ways in which the practice of CO observed by these experts exacerbates problems with accessing abortion services in Victoria; 1) by delaying or blocking access to existing services; 2) by contributing to the actual lack of providers and services.

While participants were supportive of Section 8 of the Abortion Law Reform Act as a mechanism to ensure women’s access to abortion services, all participants described situations in which the improper use of CO had negatively impacted on women seeking abortion. The negative impact was most commonly produced through the face-to-face interaction between a woman seeking abortion and a doctor with an objection, and therefore mostly remains invisible. The negative impact described in this setting ranged from an increase in guilt and discomfort for women seeking abortion, to a delay in accessing abortion, through to, in rare cases, an inability to access abortion services at all. However, a negative impact on access was also produced through individuals other than doctors claiming a CO. Both Government telephone staff and pharmacists had the potential to limit or delay access to medical abortion.

Section 8 was also perceived to reduce the availability of providers and services. The recognition of CO in Section 8 was seen by some to legitimise the practice of whole institutions opting out of abortion service provision (even where individual doctors working in that institution may not have a CO), as well as the opting out by individuals working in services which do provide abortion, thereby reducing the number of providers and compromising the capacity of the system to deliver adequate services in a timely fashion. In addition, the misrepresentation of the CO clause was used by some anti-choice groups to further their political agenda to argue against women’s right to access abortion.

Despite quantitative evidence that rates of CO are low in Australia compared to other countries [17], these qualitative results are consistent with findings reported from several surveys of US doctors; that a significant minority (15%) of practitioners who claim a CO do not adhere to obligations to refer, but instead attempt to delay or deny access [18–21]. If even a small proportion of doctors with a CO refuse to refer, this could have a significant impact on women’s access, particularly if conscientious objectors are over-represented in certain geographic areas, or see more vulnerable patients presenting later in pregnancy [40, 41].

The practice, for example, of actively seeking to delay or deter a woman from accessing abortion is clearly contrary to the “conventional compromise” or the “moderate view” of CO. The moderate view understands CO as designed to preserve the integrity of the objector by not making them complicit in what they believe is wrong. Instead in this case, the person with a CO is seeking to prevent access to a legal abortion to be performed by someone else. Evidence provided here that some doctors seek to prevent access to a legal abortion is contrary to the law and clearly outside the legitimate scope of CO. For example, the actions of one doctor who refused to refer women had significant consequences for two women seeking abortion, with a participant stating, ‘*one* [woman] *was certainly able to still seek termination, and one wasn’t, one continued* [the pregnancy]*…*’.

There were further suspicions that for some doctors who opt out of providing abortion services, objection is not based on “deeply held moral commitment” but rather on concerns about community or peer acceptance, or financial or reputational penalty. These factors were also prominent in an Australian qualitative study of factors affecting provision of medical abortion in general practice [42]. GPs who participated in the research of Dawson and colleagues are quoted as saying of medical abortion, ‘*I am not sure that’s what I want to make my business’* (p4) and *‘Somebody else can do it. I’m not interested’* (p4). Participants who were not providing medical abortion also expressed concerns about being known as the ‘abortion doctor’ and that provision would dominate their practice and change the nature of their practice or their clientele. Harries et al. [43] have called for clear guidelines to be provided to doctors in South Africa ‘in order to disentangle what is resistance to abortion provision in general, and what is conscientious objection on religious or moral grounds’ (p1). Such guidelines could be helpful in the Victorian setting to clarify the limits of the clause, and potentially reduce disingenuous claims of CO. Such guidelines would also need to be disseminated widely among general practitioners and other related service providers; they could counter misinformation about section 8 by clarifying that conscientious objectors are not required to refer to a provider of abortion services, but to refer to a practitioner without CO, who can then discuss all options. Similarly, Lee and colleagues [44] have called for guidelines to support Australian pharmacists in the provision of medical abortion.

Another significant finding of this research is concerns about the institutional ‘opting out’ of abortion provision, “*I have a really strong objection to institutional opting out because there’s nothing in the law about that and that’s wrong”*. While some have argued that an institution can have a ‘conscience’ and therefore institutional opting out may make sense as an ethical concept [45], this is far from showing that it is ethically justified, particularly in a publicly funded health care system. Participants in this study were concerned about the potentially large impact institutional opting out can have on access. It is not clear from Victorian law or policy whether it is legitimate for institutions to claim a CO, and if so, how they could address the obligation to refer women to a service able to help them, nor what constraints should be imposed on the institution’s right to make this claim. Providing clarity on this is a key issue for Government and policy makers.

### Limitations

The results of our study must be interpreted in the context of several methodological limitations. First, without comparable data from before the law reform, it is not possible to know what impact section 8 has had on the practice of doctors with a CO; we can only present a snapshot of current practice. Second, we purposively targeted Victorian health professionals with expertise in abortion service provision, therefore, we have not directly represented the views of those with a CO. Research with this group would help to confirm the nature and extent of the behaviour described by these experts. Third, being a qualitative study, our results do not give any indication as to the size of the problem, yet given the consistency in reports of the misuse of the CO clause, it is suggestive of a problem worthy of further study. Finally, the findings of this study are specific to Victoria and may not be transferable to states and territories with differing laws [46].

## Conclusion

Timely access to abortion services is dependent on both effective referral pathways and on the availability of sufficient individual providers and services to meet demand. This study shows that CO limits both factors, and therefore limits the capacity of the system to provide timely access to services. We have shown the potential limitations of implementing a “moderate” position on CO without additional training and guidelines for health practitioners and others. Further research should address policy approaches and professional and community education initiatives with the capacity to reduce barriers and improve timely access to abortion care. In support of policy change, it would be useful to determine community attitudes to the rights at play, and whether the community is willing to tolerate the negative impact that prioritising the rights of doctors has on women seeking abortion.

## Abbreviations

CO: Conscientious objection
GP: General practitioner
PBS: Pharmaceutical benefits scheme
